# Solwaric Acids A and B, Antibacterial Aromatic Acids from a Marine *Solwaraspora* sp.

**DOI:** 10.3390/md12021013

**Published:** 2014-02-14

**Authors:** Gregory A. Ellis, Thomas P. Wyche, Charles G. Fry, Doug R. Braun, Tim S. Bugni

**Affiliations:** 1 Pharmaceutical Sciences Division, University of Wisconsin-Madison, 777 Highland Avenue, Madison, WI 53705, USA; E-Mails: gaellis@wisc.edu (G.A.E.); gaellis@wisc.edu (T.P.W.); gaellis@wisc.edu (D.R.B.); 2 Department of Chemistry, University of Wisconsin-Madison, 1101 University Avenue, Madison, WI 53706, USA; E-Mail: fry@chem.wisc.edu

**Keywords:** *Trididemnum orbiculatum*, *Solwaraspora*, methicillin-resistant *Staphylococcus aureus*

## Abstract

Two novel trialkyl-substituted aromatic acids, solwaric acids A and B, were isolated from a marine *Solwaraspora* sp. cultivated from the ascidian *Trididemnum orbiculatum*. Solwaric acids A and B were isotopically labeled with U-^13^C glucose, and analysis of a ^13^C–^13^C COSY allowed for unambiguous determination of the location of the phenyl methyl group. The two novel compounds demonstrated antibacterial activity against methicillin-resistant *Staphylococcus aureus* (MRSA) and methicillin-sensitive *Staphylococcus aureus* (MSSA).

## 1. Introduction

Infectious disease continues to be a major issue, as it is the second leading cause of death worldwide [1]. Complicating the treatment of infectious disease is antibiotic resistance, which results in about $20 billion in health service costs and at least 23,000 deaths in the United States per year [2,3]. Among antibiotic resistant bacteria, methicillin-resistant *Staphylococcus aureus* (MRSA) has become a major concern for human health [4,5,6,7,8]. While the mortality percentages in methicillin-susceptible *Staphylococcus aureus* (MSSA) patients range from 5% to 28%, the mortality percentages for MRSA patients tend to be even higher, ranging from 10% to 64% [5,6,7,8]. Although ranging between studies, one study encompassing data from different European countries found an additional 80% excess mortality rate at day 30 after infection was contributed by methicillin resistance in *S. aureus* [5,6,8]. Therefore, antibiotic resistance escalates the importance for discovering new and effective antibiotics in an efficient manner.

In our pursuit of novel antibacterial compounds, we isolated two novel aromatic acids that were named solwaric acids A (**1**) and B (**2**) and the known 2,4,6-triphenyl-1-hexene (**3**) [9,10,11], from a marine *Solwaraspora* sp. (Strain WMMB329) cultivated from the ascidian *Trididemnum orbiculatum* (Van Name, 1902) [12]. To our knowledge, these are the first novel compounds reported from a *Solwaraspora* sp., although strain WMMB329 also showed 99% 16S rDNA sequence similarity to some *Micromonospora* spp. Solwaric acids A (**1**) and B (**2**) demonstrated antibacterial activity against methicillin-resistant *Staphylococcus aureus* (MRSA) and methicillin-sensitive *Staphylococcus aureus* (MSSA). Aromatic acids are somewhat rare in natural products, with most being produced by actinomycetes [13,14,15,16].

Although the solwaric acids did not present elucidation challenges typical of some natural products, such as few protons, the aromatic ring was substituted only with carbon-based substituents. As a result, the chemical shifts of the protons presented challenges with respect to unambiguously determining the location of the methyl group. HMBC correlations were not conclusive and ^13^C calculations using density functional theory were questionable due to the small differences between two potential structures. Therefore, solwaric acids A (**1**) and B (**2**) were isotopically enriched with U-^13^C glucose, which allowed for acquisition of a ^13^C–^13^C COSY and unambiguous assignment of the aromatic methyl group location. While the gCOSY requires isotopic enrichment, the method is still economically feasible, and can be made even more so by the use of ^13^C-optimized cryoprobes, which drastically reduce the amount of compound needed and therefore the amount of U-^13^C glucose required. We have more broadly explored the ^13^C gCOSY as a rapid route for establishing carbon connectivity, and it appears broadly useful for a broad range of bacterially produced natural products. Overall, we have found this method to be highly promising.

## 2. Results and Discussion

### 2.1. Bacterial Strain Selection and Structure Elucidation

**Figure 1:**
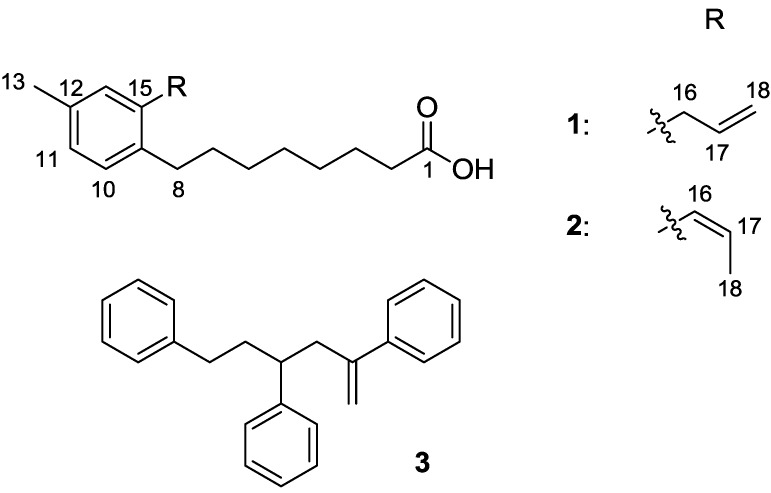
Structures of **1**–**3**.

Strain WMMB329 was investigated due to the lack of chemistry described in the literature from *Solwaraspora* spp. as well as antibacterial activity from initial screening of a crude extract. After fermentation and isolation of the bioactive compounds, WMMB329 was found to produce solwaric acids A (**1**) and B (**2**), as well as the known 2,4,6-triphenyl-1-hexene (**3**) (Figure 1).

HRMS supported the molecular formula of C_18_H_26_O_2_ for both solwaric acid A (**1**) and B (**2**). The structures of solwaric acids A (**1**) and B (**2**) were determined by analysis of ^1^H and ^13^C NMR data (Table 1, Supplementary Figures S1–S16). Since the assignment of solwaric acid A (**1**) and B (**2**) was confirmed by direct ^13^C–^13^C correlation experiments, the discussion of elucidation has been eliminated with the exception of the olefin at C-16 and C-17 in solwaric acid B (**2**), which was determined to be *Z* based on the vicinal coupling constant (^3^*J*_H_ 11.4) [17]. The presence of a methyl group (C-13) attached to the phenyl ring was indicated by HMBC correlations to three aromatic carbon atoms, but it could not be determined unambiguously relative to the other aromatic carbons. The major problem was that two protons in the aromatic ring (H-11 and H-14) were at nearly identical chemical shifts. Additionally, C-9 and C-15 were at nearly identical chemical shifts. Therefore, the HMBC correlations supported two possible arrangements for C-10 through C-14 relative to positions 9 and 15. To further complicate matters, the close chemical shifts of H-11 and H-14 made it difficult to justify assignment based on NOE. Finally, DFT calculations showed differences in the calculated ^13^C shifts that were within what we have observed as typical experimental error (*i.e.*, not overly conclusive). Direct ^13^C–^13^C correlations, however, would solve the problem. Consequently, we evaluated the use of ^13^C-labeling of the two compounds using U-^13^C glucose and subsequent acquisition of a ^13^C–^13^C gCOSY to address this issue.

**Table 1:** ^1^H and ^13^C NMR data for **1** and **2** (600 MHz for ^1^H, 150 MHz for ^13^C, CDCl_3_).

Position	1			2		COSY	HMBC
	δ_C_, mult.	δ_H_ (*J* in Hz)		δ_C_, mult.	δ_H_ (*J* in Hz)		
1	180.1, C			180.5, C			
1-OH					9.34, s		
2	34.4, CH_2_	2.33, t (6.6)		34.8, CH_2_	2.29, t (6.6)	3	3, 4
3	24.9, CH_2_	1.60, m		25.0, CH_2_	1.59, m	2, 4	2, 4
4	29.2, CH_2_	1.30, m		29.3, CH_2_	1.30, m	3	
5	29.4, CH_2_	1.30, m		29.4, CH_2_	1.30, m		
6	29.9, CH_2_	1.30, m		29.6, CH_2_	1.30, m	7	
7	31.3, CH_2_	1.51, m		30.9, CH_2_	1.48, m	8	9
8	32.5, CH_2_	2.52, t (7.6)		33.2, CH_2_	2.50, t (7.6)	7	6, 9, 10
9	137.9, C			138.1, C			
10	129.4, CH	7.02, d (7.2)		129.0, CH	7.04, d (7.2)	11	8, 12
11	127.2, CH	6.95, d (7.2)		127.6, CH	6.96, d (7.2)	10	14
12	135.5, C			134.7, C			
13	21.2, CH_3_	2.27, s		21.2, CH_3_	2.30, s		11, 12, 14
14	130.4, CH	6.94, s		130.3, CH	6.97, s		9, 13
15	137.5, C			136.1, C			
16	37.2, CH_2_	3.33, br d (6.4)		129.1, CH	6.47, br d (11.4)	17	12, 14, 18
17	137.7, CH	5.93, tdd (6.4, 10.1, 16.9)		126.9, CH	5.77, dq (11.4, 7.0)	16, 18	18
18	115.7, CH_2_	5.04, tdd (1.7, 1.7, 10.1)		14.6, CH_3_	1.71, dd (1.8, 7.0)	17	16, 17
		5.00, tdd (1.7, 1.7, 16.9)					

### 2.2. ^13^C–^13^C gCOSY

NMR experiments, such as the 2D INADEQUATE, have been used for direct determination of carbon-carbon connectivity within natural products [18,19]. Even with cryoprobes, the INADEQUATE can present difficulties due to sample solubility since it requires a fairly concentrated sample. Therefore, we decided to evaluate a ^13^C–^13^C gCOSY, which would require a shorter minimum phase cycle than the INADEQUATE, could be easily implemented, and could be easily interpreted. In particular, calculations suggested that the standard two-pulse gCOSY implemented on NMR spectrometers for ^1^H experiments could be used for ^13^C–^13^C with no modifications to the pulse program. In fact, after adjusting for ^13^C and spectral widths, a theoretical sampling of coupling constants in the range 30 to 60 Hz would produce maximum polarization transfer at 33 to 17 ms, respectively. Additionally, S/N for *J*_CC_ = 60 Hz would be maximized with 450 increments—though smaller values (e.g., 256) could be used without much loss in sensitivity—resulting in the 2D resolution of 0.55 ppm (Supplementary Figure S17). The combination of easily optimizing for a range of ^13^C–^13^C coupling constants combined with the fact that the gCOSY does not require ^13^C inversion pulses and that the experiment could be immediately implemented by others with no requirement for pulse programming made the simple gCOSY an encouraging prospect (Supplementary Figures S18 and S19). The only drawback was that the ^13^C–^13^C COSY experiment requires isotopic enrichment. To address this problem, we tested the feasibility of ^13^C incorporation of microbial-derived natural products in conjunction with evaluating the feasibility of the ^13^C–^13^C gCOSY.

**Figure 2:**
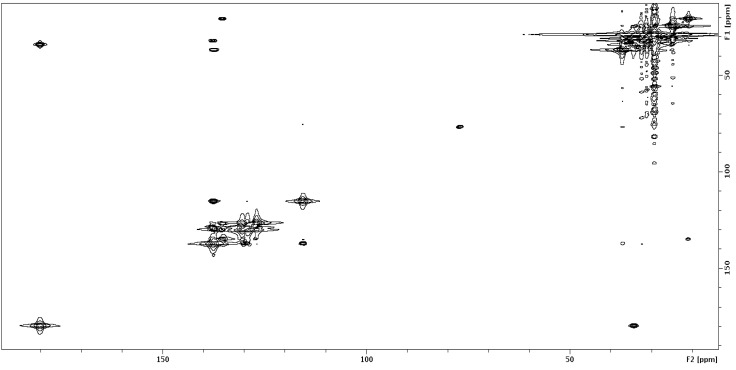
^13^C–^13^C COSY of ^13^C-labeled **1**.

To increase the ^13^C abundance, fermentation of WMMB329 in 1 L ASW-A using uniformly ^13^C-labeled glucose (10 g/L ASW-A) and subsequent purification yielded solwaric acids A (**1**) and B (**2**) with about 35% ^13^C incorporation (as evidenced by MS isotopic distribution). The ^13^C–^13^C gCOSY (Figure 2) was acquired in two hours on 18 µmoles (105 µM) solwaric acid A (**1**) and allowed for the methyl group (C-13) to be unambiguously assigned as attached to C-12; likewise, ^13^C–^13^C COSY correlations were seen between C-12 and C-14, as well as C-14 and C-15. Analysis of the ^13^C–^13^C COSY also confirmed the carbon connectivity throughout the rest of the structure (Supplementary Figure S20). While the ^13^C–^13^C gCOSY was acquired on 5 mg of solwaric acid A (**1**), the experiment can be effectively acquired at considerably lower concentrations. The exact concentration is dependent on the level of ^13^C incorporation and the spectrometer used, but our initial studies suggest that a ^13^C–^13^C gCOSY can be effectively acquired on low µM concentrations in less than 24 h.

We have also tested to see if this approach would be more broadly applicable to other types of natural products. For example, we have produced two novel ^13^C-labeled peptides from marine-derived bacteria, the structures of which will be reported in a subsequent publication. For complicated structures, this method will drastically reduce elucidation time, which is currently a bottleneck in natural products discovery. Also, recent work by Williamson and Martin has shown that ^13^C–^13^C coupling constants can aid in establishing configuration [20]. Even modest levels of ^13^C incorporation would be helpful to apply ^13^C–^13^C coupling constants for help in structure elucidation.

Additionally, ^13^C-labeling and acquisition of a ^13^C–^13^C COSY can be useful for analyzing crude extracts or mixtures of compounds. We have demonstrated this approach by acquiring a ^13^C–^13^C COSY on the extract from the CHCl_3_ partition of WMMB329 (Supplementary Figure S21). The CHCl_3_ partition was the first step of purification of the crude extract, and therefore, contained a mixture of compounds. Acquisition of the ^13^C–^13^C COSY on ^13^C-labeled extract allows for separation of the chemistry on a large sweep width (~200 ppm) and elimination of most media components—which are unlabeled—from the spectrum. Although there may still be a mixture of several labeled compounds, this method could rapidly provide carbon-carbon connectivity for portions of the structures, which could be helpful for dereplication. Therefore, ^13^C-labeling of natural products can be a valuable tool for structure elucidation, as well as other areas of natural product research.

### 2.3. Biological Activity

Solwaric acids A (**1**) and B (**2**) showed antibacterial activity and were more potent against gram positive bacteria (Table 2). Vancomycin and gentamicin were used as a positive control in assays with gram positive and gram negative bacteria, respectively. The known 2,4,6-triphenyl-1-hexene (**3**) (Supplementary Figures S22–S24) demonstrated no antibacterial activity.

**Table 2:** Minimum Inhibitory Concentration (µg/mL) of **1**–**3**.

	MRSA	MSSA	*E. coli*	*P. aeruginosa*
**1**	32	64	128	128
**2**	32	64	128	128
**3**	>128	>128	>128	>128

## 3. Experimental Section

### 3.1. General Experimental Procedures

Optical rotations were measured on a Perkin–Elmer 241 Polarimeter. UV spectra were recorded on an Aminco/OLIS UV-Vis spectrophotometer. IR spectra were measured with a Bruker Equinox 55/S FT–IR spectrophotometer. NMR spectra were obtained in CDCl_3_ with a Bruker Avance 600 MHz spectrometer equipped with a 1.7 mm ^1^H{^13^C/^15^N} cryoprobe, a Bruker Avance 500 MHz spectrometer equipped with a ^13^C/^15^N{^1^H} cryoprobe, and a Varian Unity-Inova 500 MHz spectrometer. HRMS data were acquired with a Bruker MaXis™ 4G QTOF mass spectrometer. RP HPLC was performed using a Shimadzu Prominence HPLC system and a Phenomenex Luna C18 column (250 × 10 mm, 5 µm), as well as a Gilson Preparative HPLC and Phenomenex Gemini C18 column (250 × 30 mm, 5 µm).

### 3.2. Biological Material

Ascidian specimens were collected on 11 October 2010, in the Florida Keys (24°37.4873′, 81°27.443′). Identification was confirmed by Shirley Parker-Nance. A voucher specimen (FLK10-5-1) for *Trididemnum orbiculatum* (Van Name, 1902) [12] is housed at the University of Wisconsin-Madison. For cultivation, a sample of ascidian (1 cm^3^) was rinsed with sterile seawater, macerated using a sterile pestle in a micro-centrifuge tube, and dilutions were made in sterile seawater, with vortexing between steps to separate bacteria from heavier tissues. Dilutions were separately plated on three media: ISP2 supplemented with artificial seawater [21], R2A [22], and M4 [23]. Each medium was supplemented with 50 µg/mL cycloheximide and 25 µg/mL nalidixic acid. Plates were incubated at 28 °C for at least 28 days, and strain WMMB329 was purified from an M4 isolation plate.

### 3.3. Sequencing

16S rDNA sequencing was conducted as previously described [24]. WMMB329 was identified as a *Solwaraspora* sp. and demonstrated 99% sequence similarity to *Solwaraspora* sp. UMM486 (accession number AY552774) and 99% sequence similarity to *Micromonospora* sp. S3-1 (accession number AB645957). The 16S sequence for WMMB329 was deposited in GenBank (accession number KC856821).

### 3.4. Fermentation, Extraction, and Isolation

One 10 mL seed culture (25 × 150 mm tube) in medium modified ASW-A (5 g soluble starch, 10 g glucose, 5 g peptone, 5 g yeast extract per liter of 50%/50% artificial seawater/diH_2_O) were inoculated with strain WMMB329 and shaken (200 RPM, 28 °C) for fifteen days. For fermentation, 500 mL baffled flasks (2 × 100 mL) containing ASW-A (20 g soluble starch, 10 g glucose, 5 g peptone, 5 g yeast extract, 5 g CaCO_3_ per liter of artificial seawater) were inoculated with 4 mL seed culture and were incubated (200 RPM, 28 °C) for fourteen days. Two-liter flasks (15 × 500 mL) containing medium ASW-A with Diaion HP20 (7% by weight) were inoculated with 5 mL from the 100 mL culture and shaken (200 RPM, 28 °C) for ten days. Filtered HP20 and cells were washed with H_2_O and extracted with acetone. The acetone extract (7.1 g) was subjected to liquid-liquid partitioning using 90%/10% MeOH/H_2_O and hexanes (1:1) followed by liquid-liquid partitioning of the previous aqueous layer using 70%/30% MeOH/H_2_O and CHCl_3_ (1:1). The hexanes partition (287 mg) was fractionated by Sephadex LH20 column chromatography (2.5 × 40 cm, CHCl_3_:MeOH, 1:1). Fractions containing 1–3 were subjected to RP HPLC (70/30% to 90/10% ACN/H_2_O with H_2_O containing 0.1% acetic acid, 25 min, 25 mL/min followed by 90/10% to 100/0% of same solvents, 3 min, 25 mL/min, and a hold at 100/0% of same solvents) using a Phenomenex Gemini C18 column (250 × 30 mm, 5 µm), yielding 1 (15.4 mg, *t*_R_ 18.0 min), 2 (13.8 mg, *t*_R_ 19.5 min), and 3 (4.9 mg, *t*_R_ 31.5 min). For ^13^C incorporation, the same procedure was used with two-liter flasks (2 × 500 mL) containing medium ASW-A (U-^13^C-glucose substituted for unlabeled glucose). Yields for solwaric acids A (**1**) and (**2**) were approximately the same during fermentation with U-^13^C glucose as with unlabeled glucose.

Solwaric acid A (**1**). colorless solid; UV (MeOH) λ_max_ (log ε) 204 (2.77), 218 (2.49), 271 (1.27) nm; IR (ATR) ν_max_ 2928, 2856, 1707, 1412, 1217, 994, 911, 819, 755 cm^−1^; ^1^H and ^13^C NMR (Table 1); HRMS [M + Na]^+^*m*/*z* 297.1836 (calcd. for C_18_H_26_O_2_Na^+^, 297.1825).

Solwaric acid B (**2**). colorless solid; UV (MeOH) λ_max_ (log ε) 208 (2.79), 238 (2.48), 281 (1.29) nm; IR (ATR) ν_max_ 2928, 2856, 1708, 1410, 1217, 820, 755, 707, 667 cm^−1^; ^1^H and ^13^C NMR (Table 1); HRMS [M + Na]^+^*m*/*z* 297.1838 (calcd. for C_18_H_26_O_2_Na^+^, 297.1825).

### 3.5. Antibacterial Assay

Solwaric acid A (**1**) and B (**2**) and 2,4,6-triphenyl-1-hexene (**3**) were tested for antibacterial activity against MSSA (ATCC #29213), MRSA (ATCC #33591), *E. coli* (ATCC #25922), and *P. aeruginosa* (ATCC #27853), and MICs were determined using a dilution antimicrobial susceptibility test for aerobic bacteria [25]. Solwaric acids A (**1**) and B (**2**) and 2,4,6-triphenyl-1-hexene (**3**) were dissolved in DMSO, serially diluted to 10 concentrations (0.25–128 µg/mL), and tested in a 96-well plate. Vancomycin was used as a control and exhibited an MIC of 1 µg/mL against MSSA and 1 µg/mL against MRSA. Gentamicin was used as a control and exhibited an MIC of 4 µg/mL against *E. coli* and 4 µg/mL against *P. aeruginosa*. Solwaric acids A (**1**) and B (**2**) and 2,4,6-triphenyl-1-hexene (**3**) were tested in triplicate, and vancomycin and gentamicin was tested in triplicate. Six untreated media controls were included on each plate. The plates were incubated at 33 °C for 18 h. The MIC was determined as the lowest concentration that inhibited visible growth of bacteria.

## 4. Conclusions

We reported the isolation and structure elucidation of two novel trialkyl-substitued aromatic acids, solwaric acids A (**1**) and B (**2**), and the known 2,4,6-triphenyl-1-hexene (**3**). The novel compounds demonstrated antibacterial activity against methicillin-resistant *Staphylococcus aureus* (MRSA) and methicillin-sensitive *Staphylococcus aureus* (MSSA). Solwaric acid A (**1**) and B (**2**) were enriched with ^13^C-labeled glucose that allowed for the acquisition of a ^13^C–^13^C COSY and unambiguous assignment of the methyl group location on the phenyl ring. While this example utilized ^13^C-labeling to determine carbon connectivity for one challenging portion of the structure, this method could be valuable for molecules with multiple tetrasubstituted centers, which make structure elucidation by standard NMR experiments more challenging. Hence, ^13^C incorporation and subsequent acquisition of a ^13^C–^13^C COSY—aided by the increasing sensitivity of NMR spectrometers—could drastically reduce the time for structure determination of microbial-derived natural products, including peptides and terpenes. Compared to the cost and time involved with other methods such as computer assisted structure determination, labeling microbial natural products offers a cost effective solution while providing high confidence in the proposed structure.

## Supplementary Files

Supplementary File 1 Supplementary Information (PDF, 668 KB)
