# Supplementary material for: Solwaric Acids A and B, Antibacterial Aromatic Acids from a Marine Solwaraspora sp
Source: Mar Drugs. 2014 Feb 14;12(2):1013–22. doi: 10.3390/md12021013 (PMC3944528; doi:10.3390/md12021013)
Supplement: Supplementary File 1 — Supplementary Information (PDF, 668 KB) [file marinedrugs-12-01013-s001.pdf]

Supplementary Information

Figure S1. ^{1}H NMR (600 MHz, $CDCl_3$) of **1**.

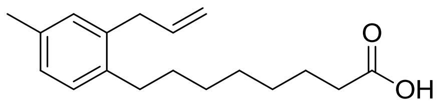

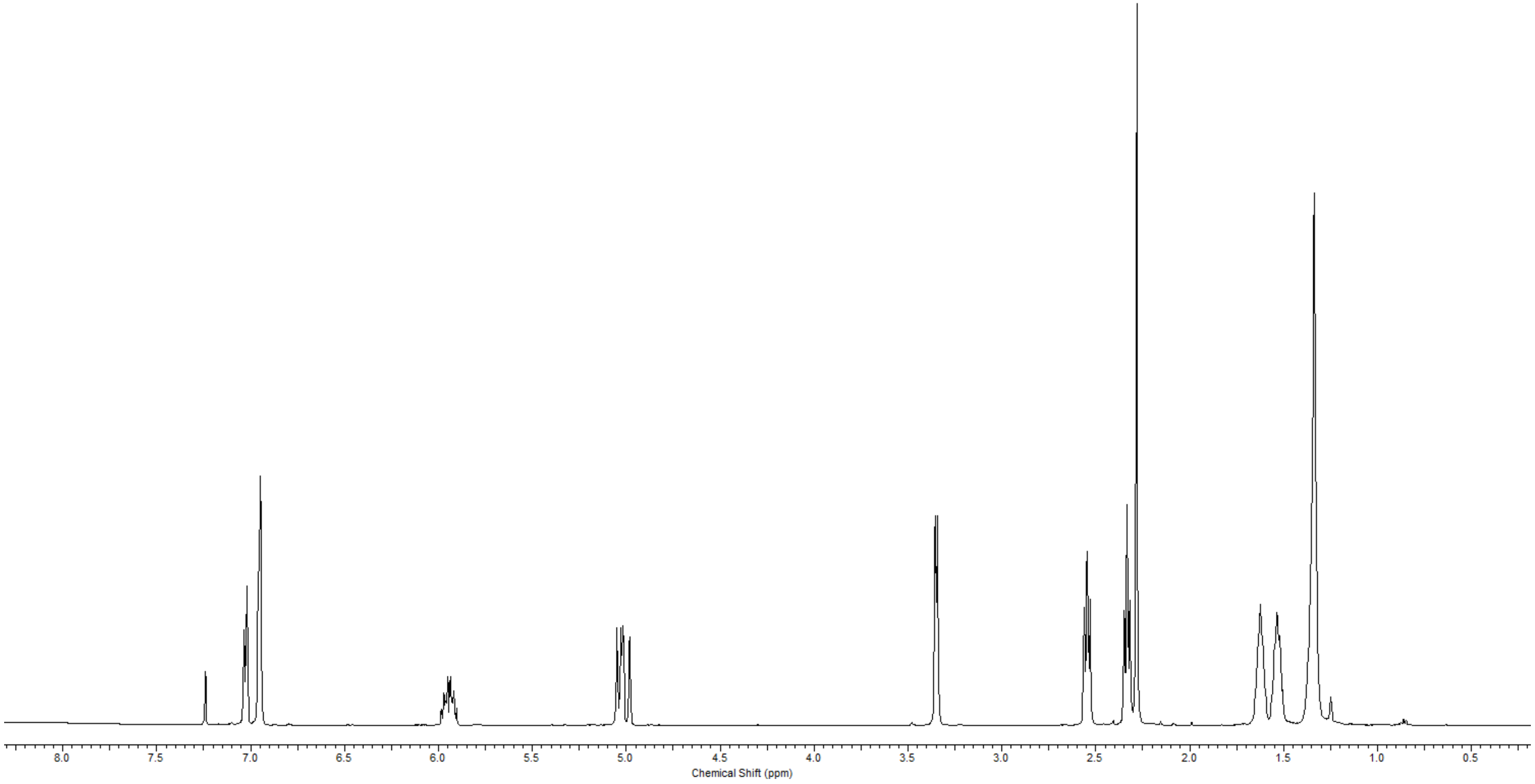

Figure S2. ^{13}C NMR (125 MHz, $CDCl_3$) of **1**.

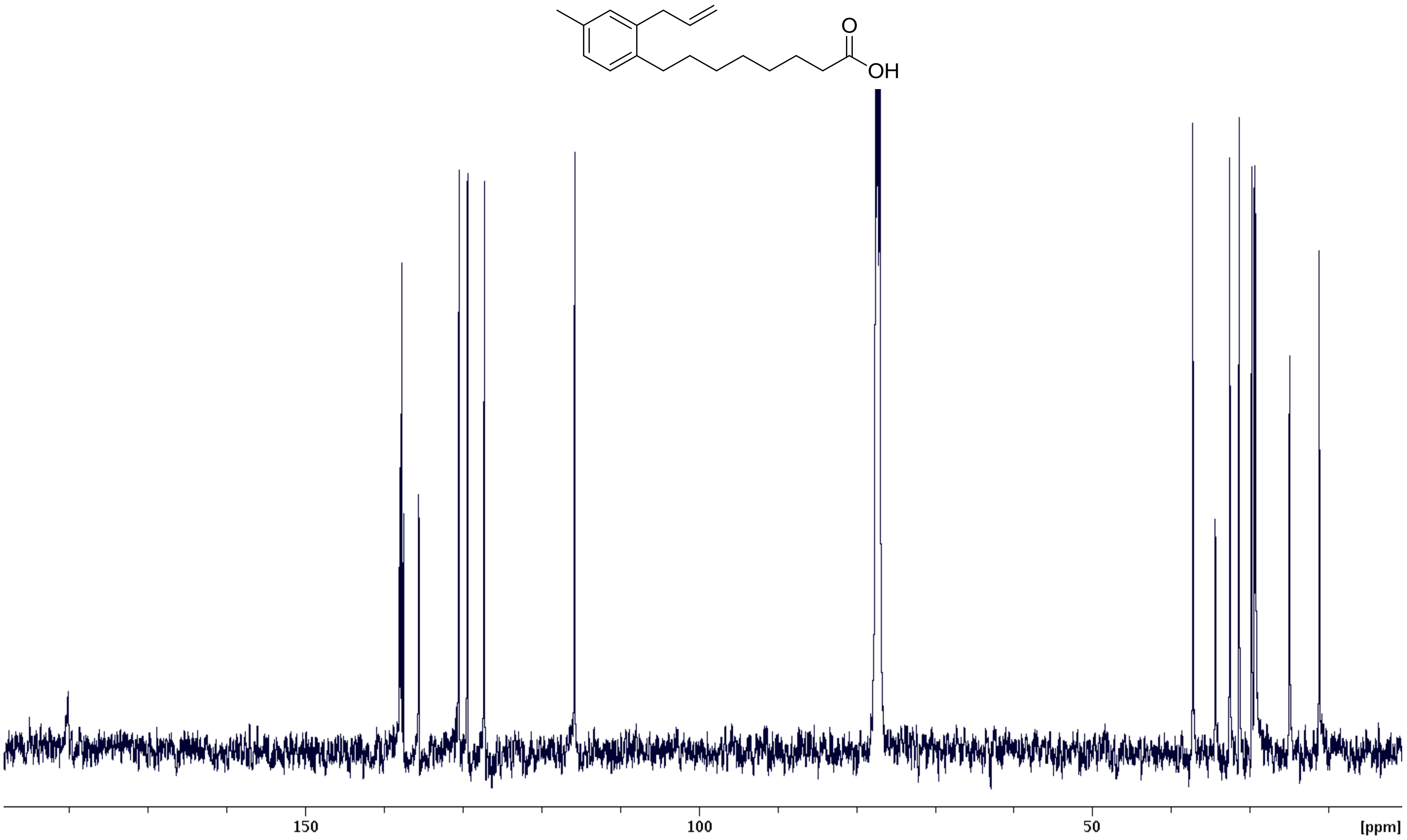

Figure S3. gCOSY (600 MHz, $CDCl_3$) of **1**.

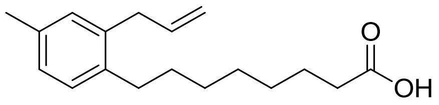

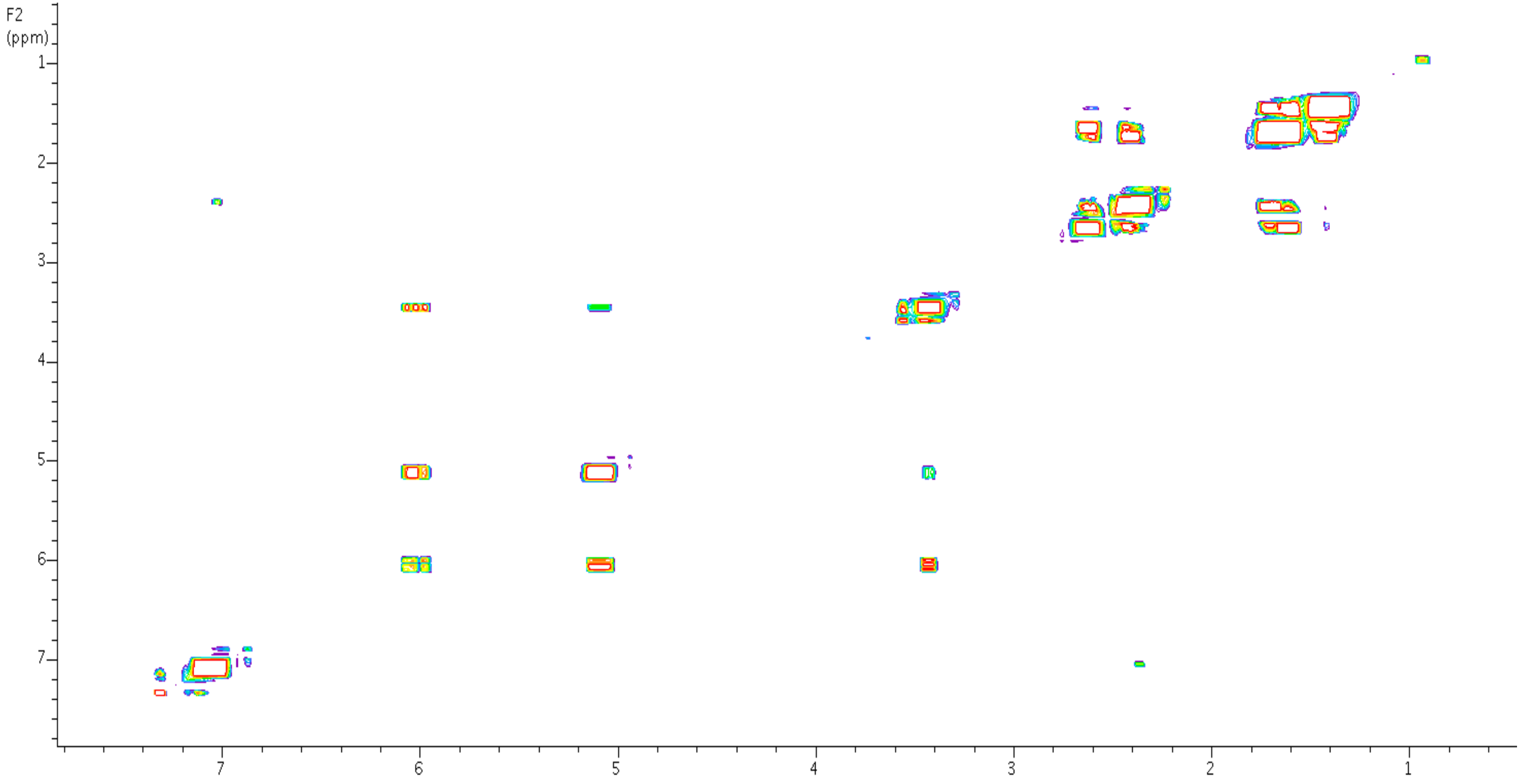

Figure S4. gHSQC (600 MHz, $CDCl_3$) of **1**.

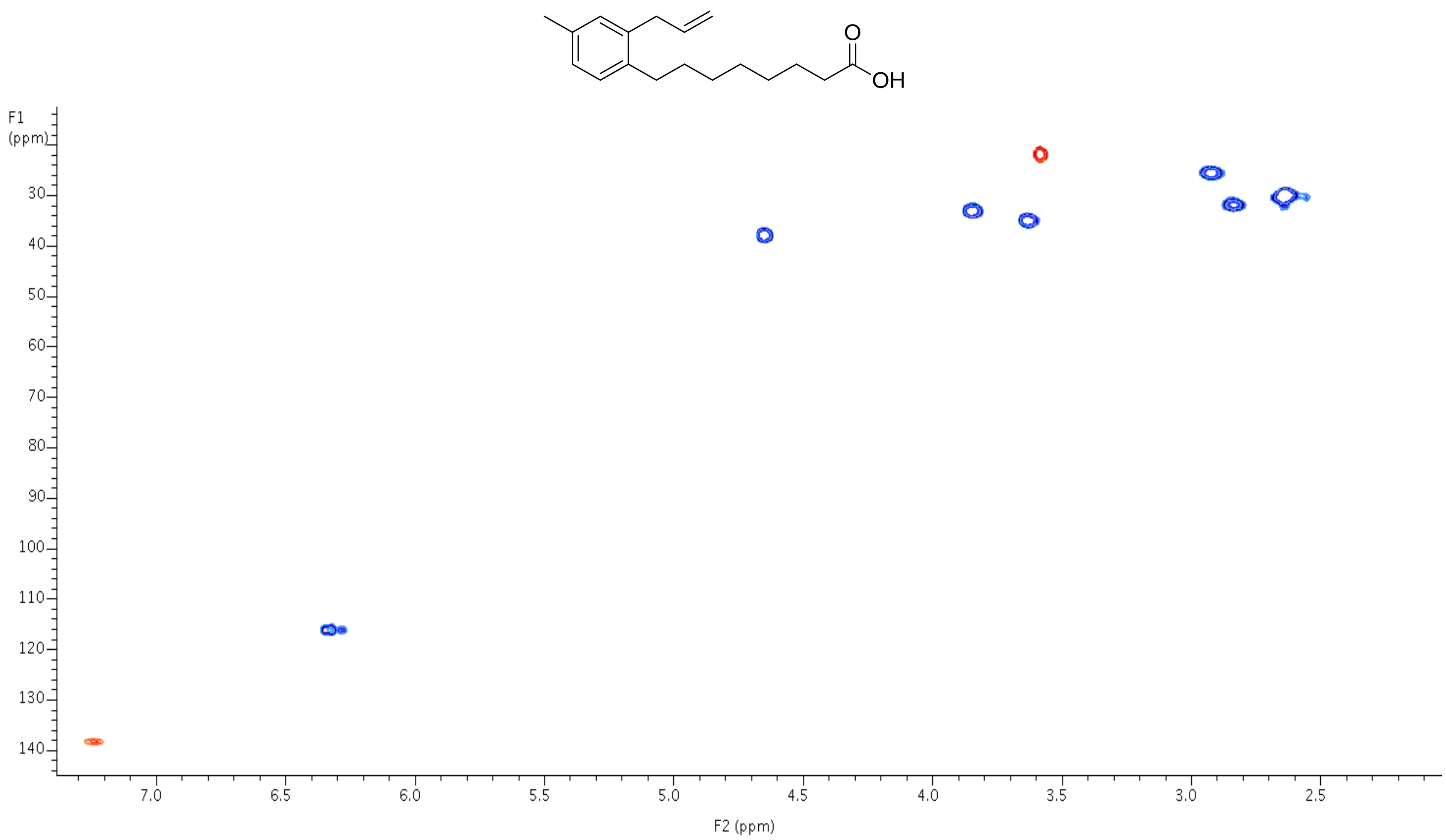

Figure S5. gHMBC (600 MHz, $CDCl_3$) of **1**.

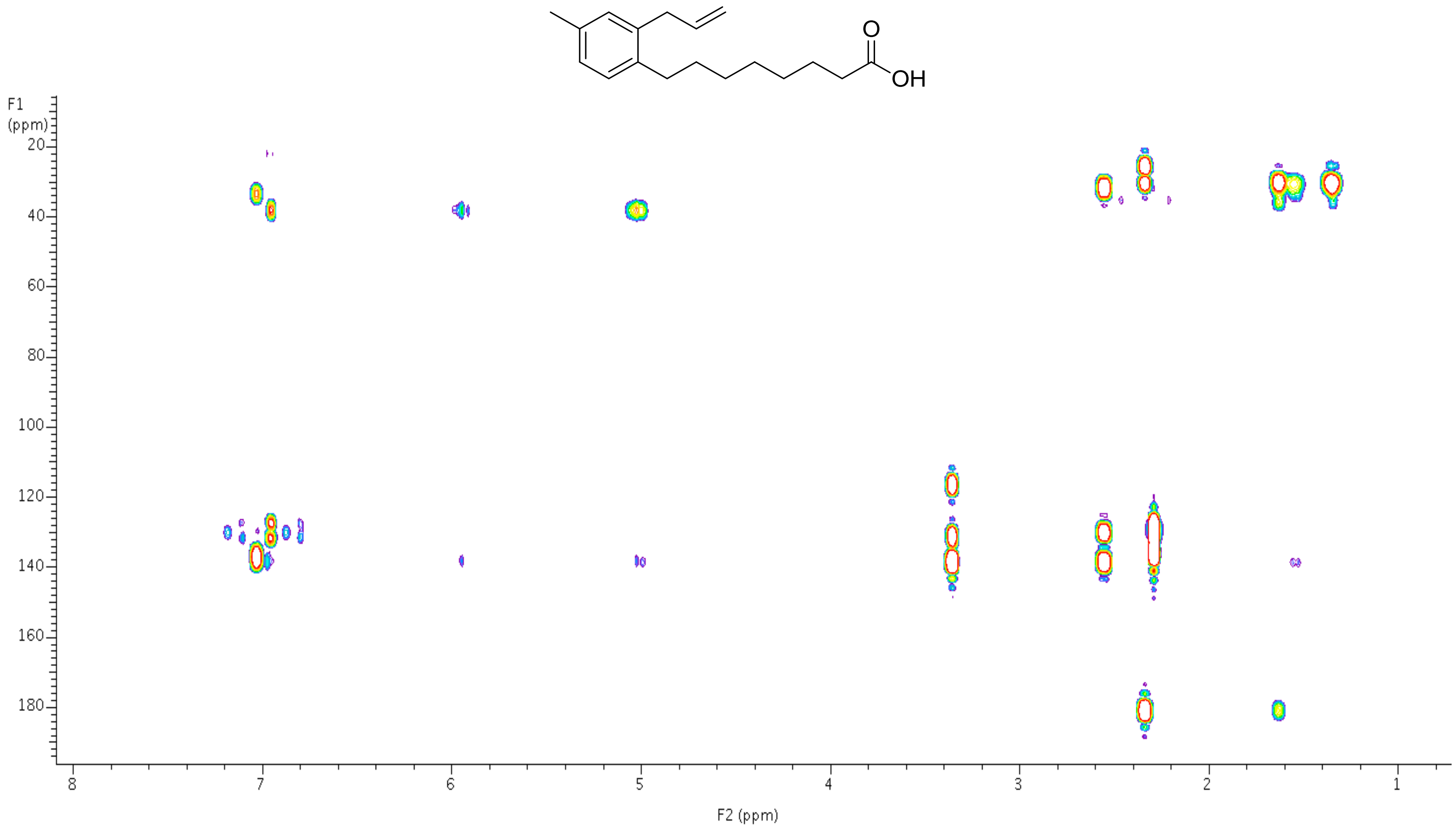

Figure S6. ^{13}C NMR (125 MHz, $CDCl_3$) of ^{13}C-labeled **1**.

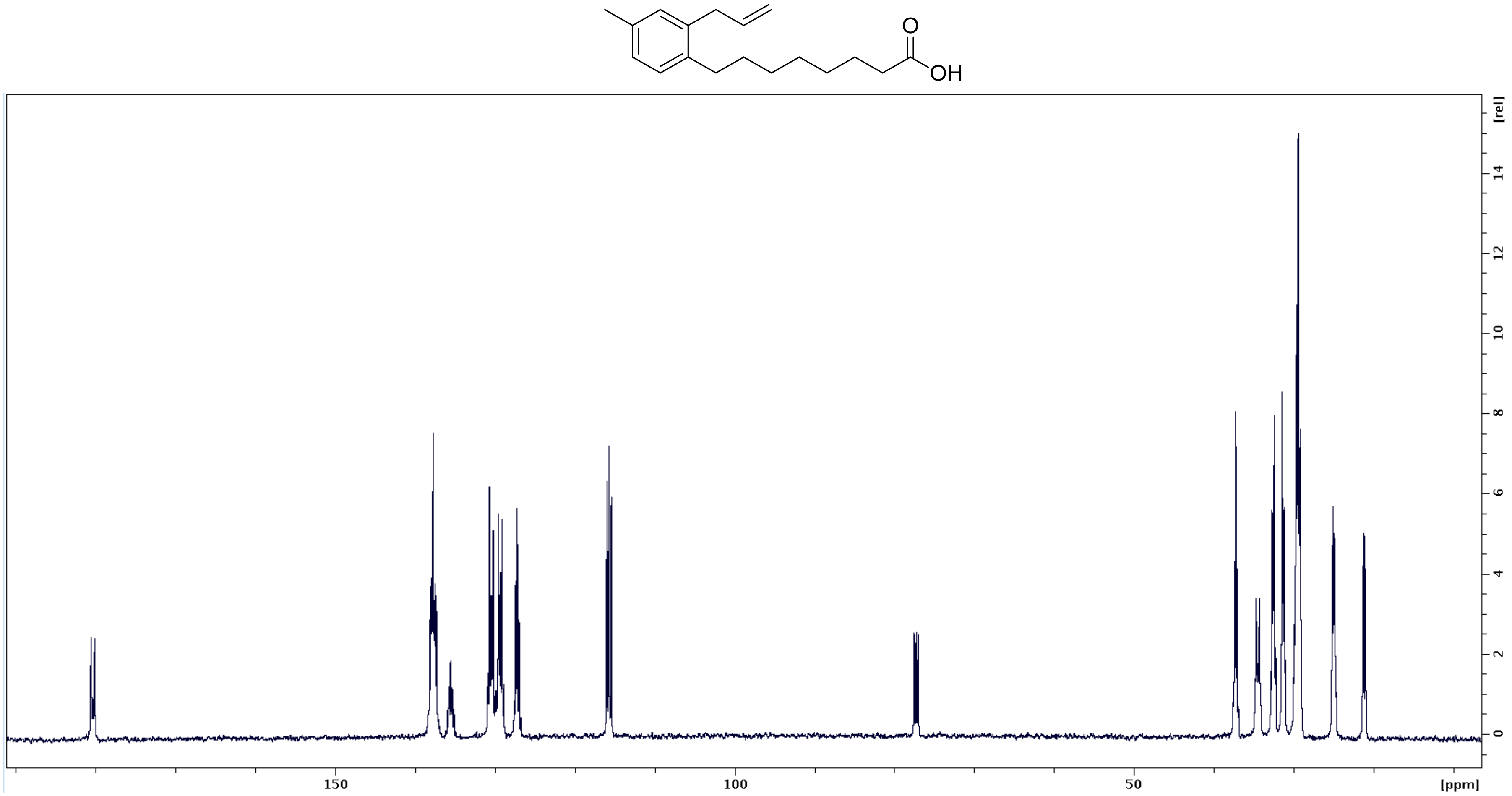

Figure S7. ^{13}C-^{13}C COSY (125 MHz, $CDCl_3$) of ^{13}C-labeled **1**.

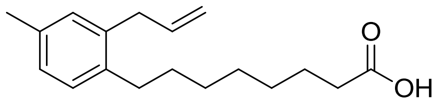

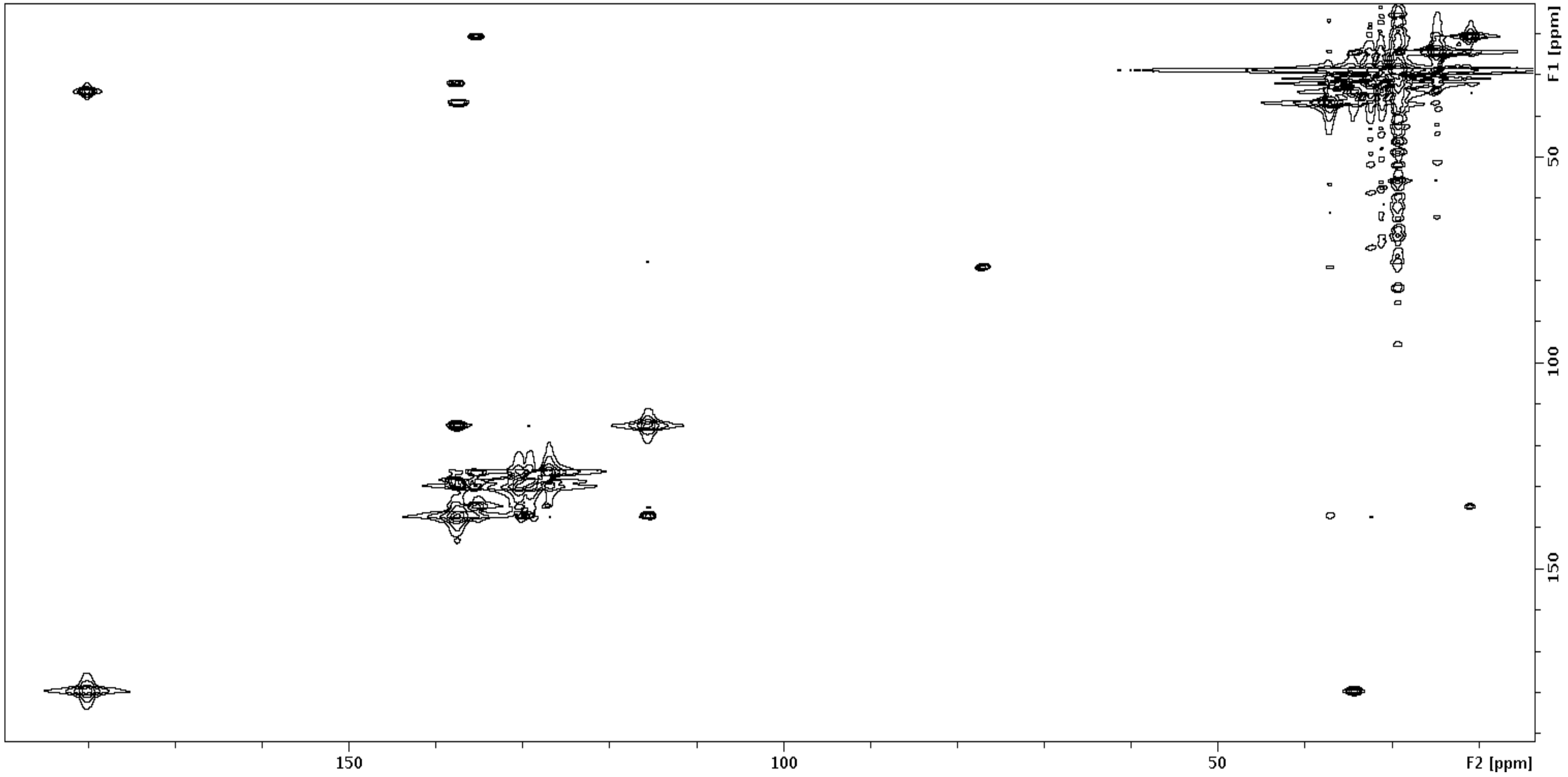

Figure S8. HRMS of **1**.

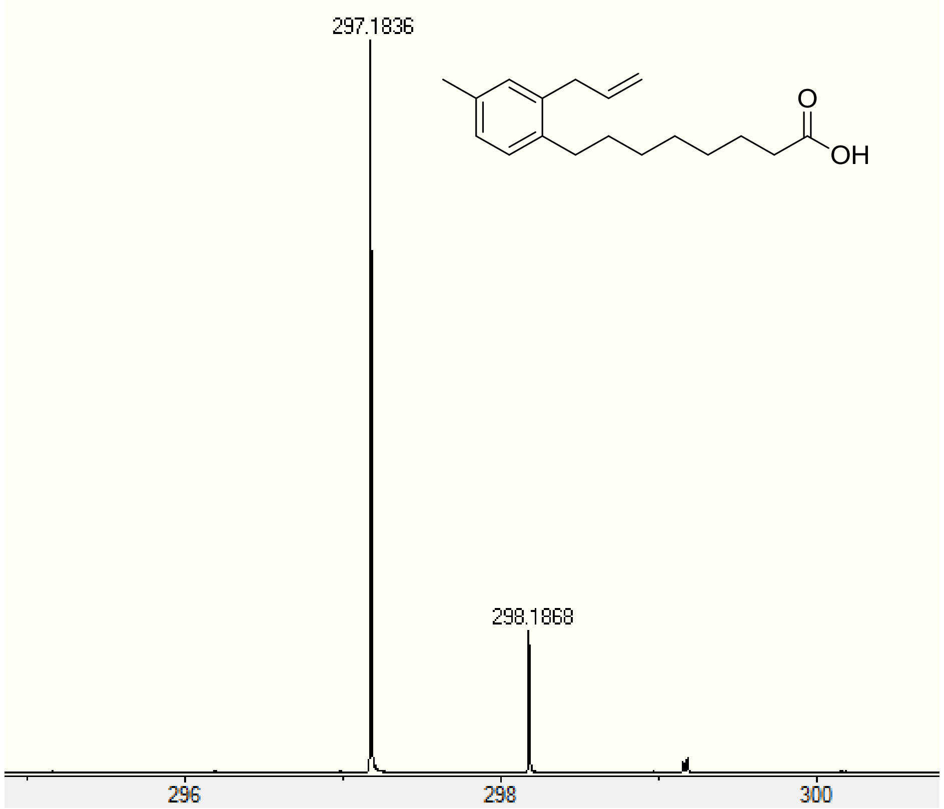

Figure S9. ^{1}H NMR (600 MHz, $CDCl_3$) of **2**.

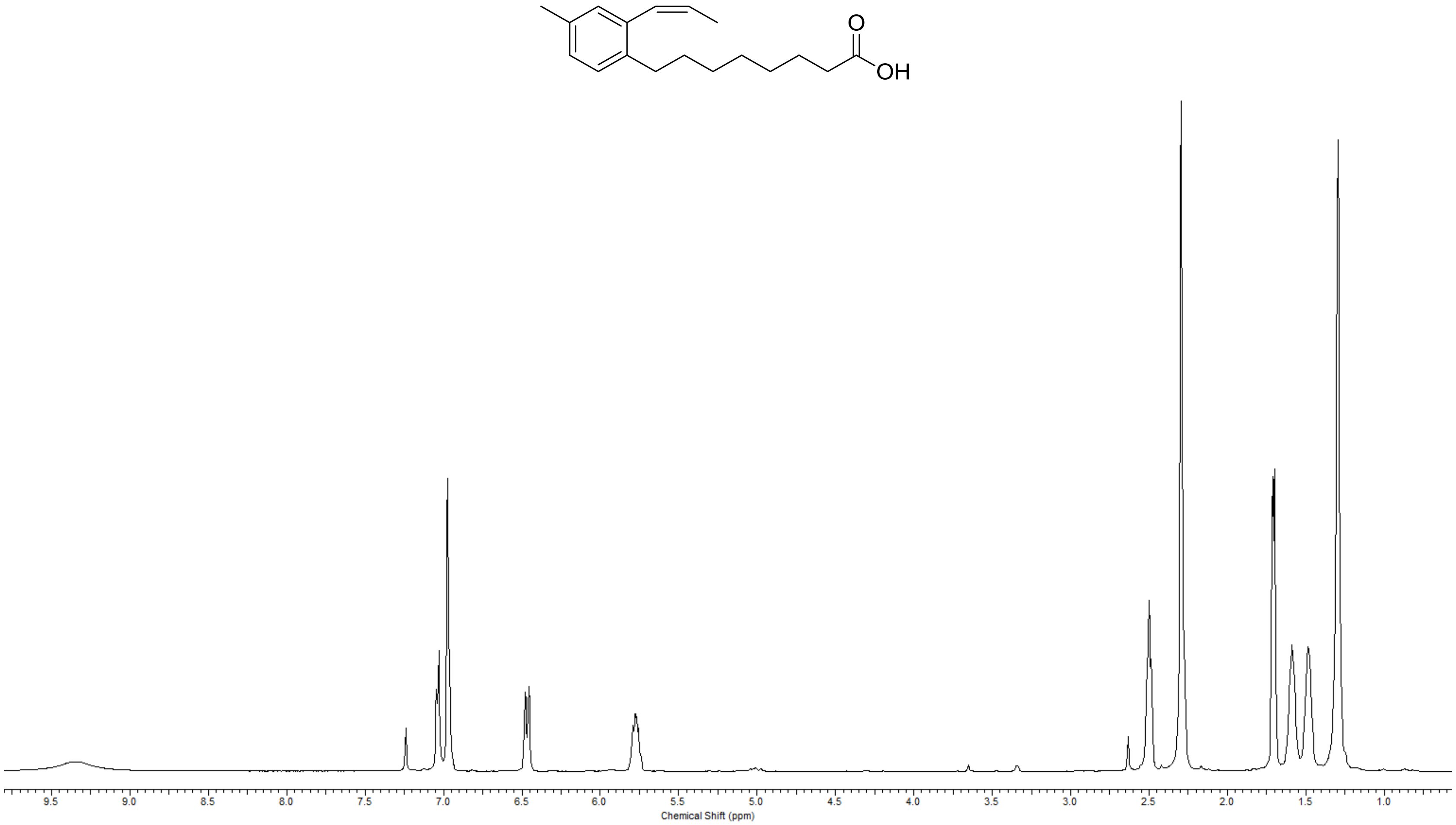

Figure S10. ^{13}C NMR (125 MHz, $CDCl_3$) of **2**.

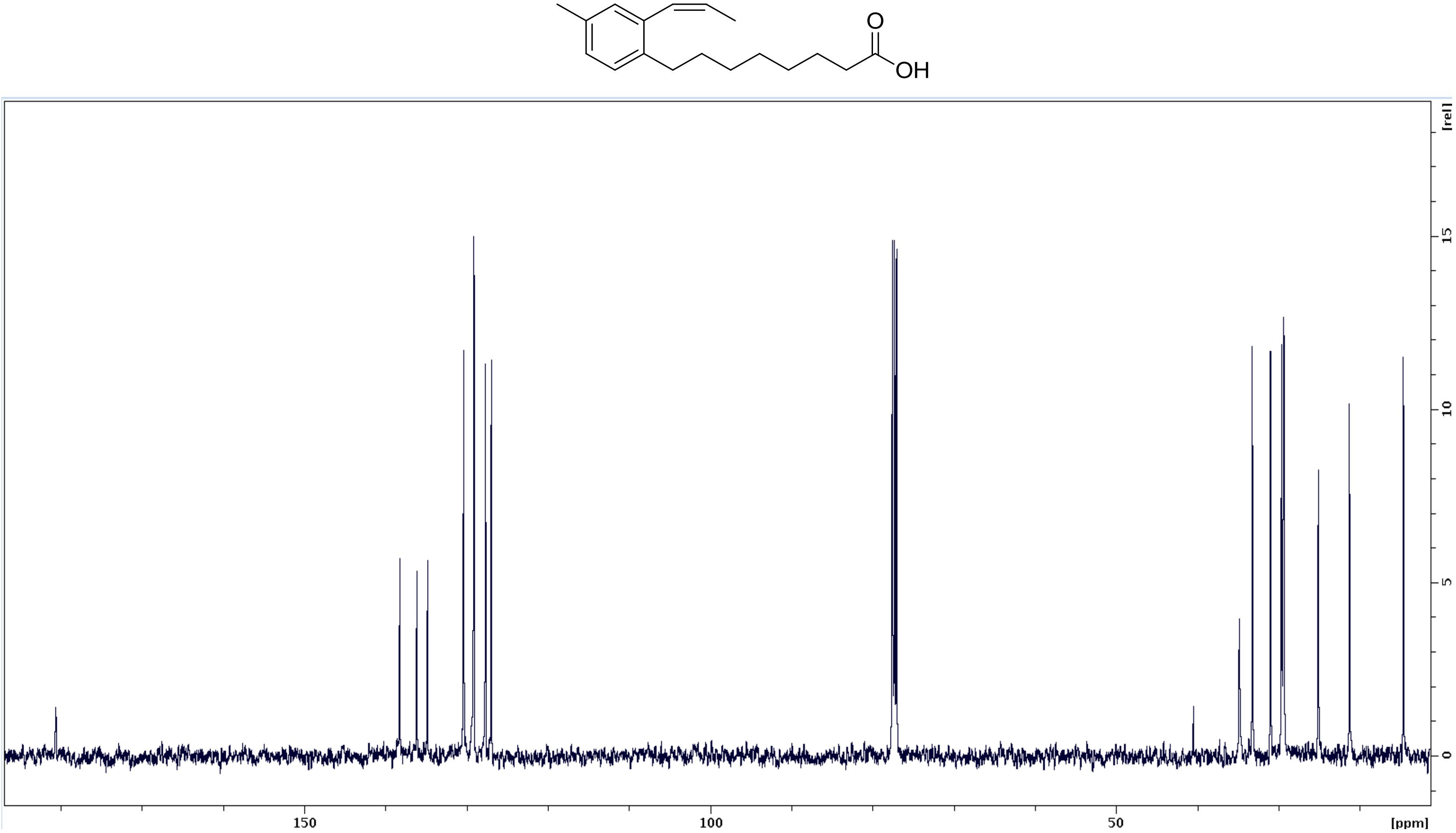

Figure S11. gCOSY (600 MHz, $CDCl_3$) of **2**.

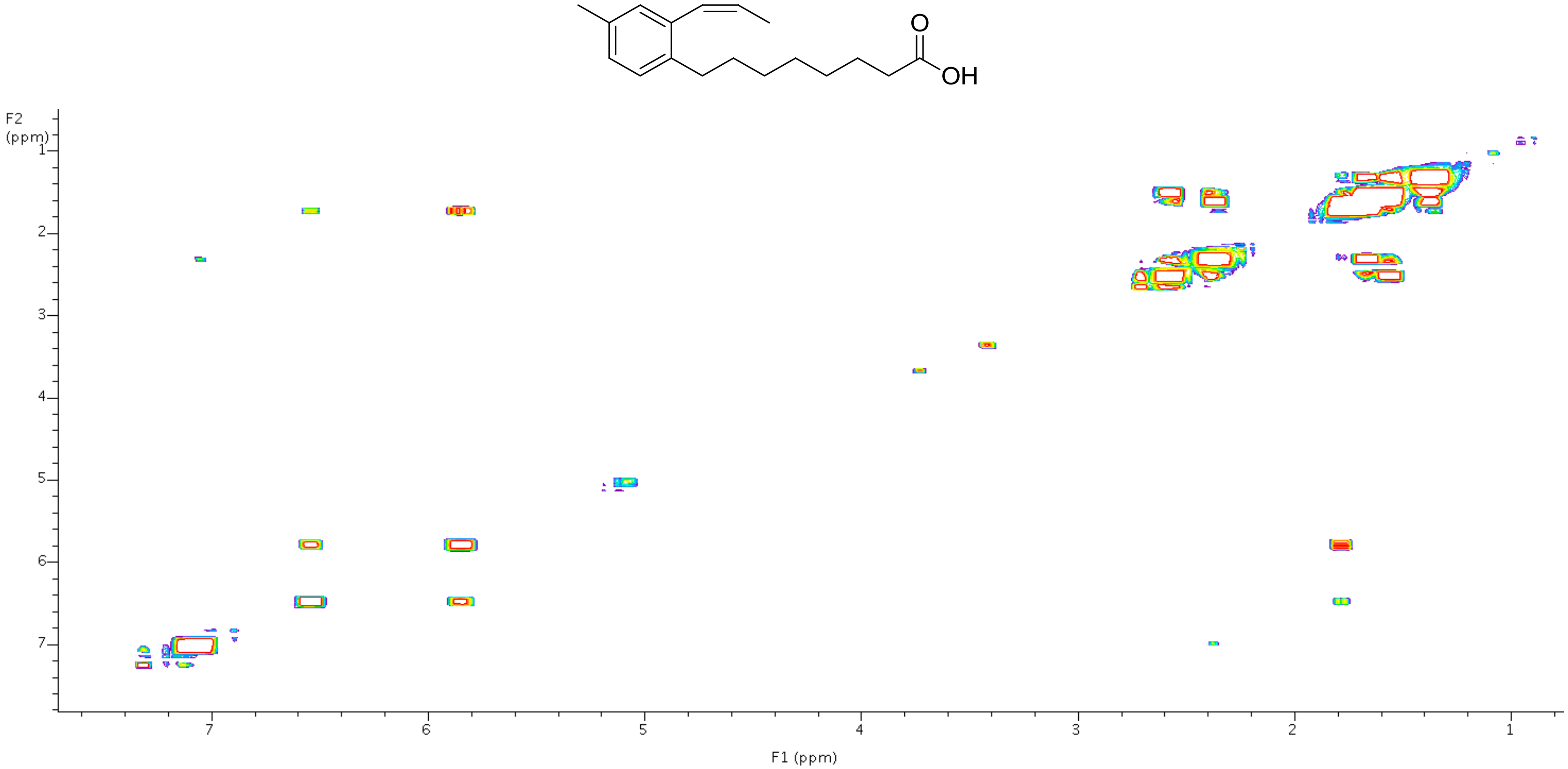

Figure S12. gHSQC (600 MHz, $CDCl_3$) of **2**.

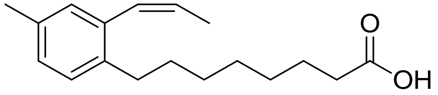

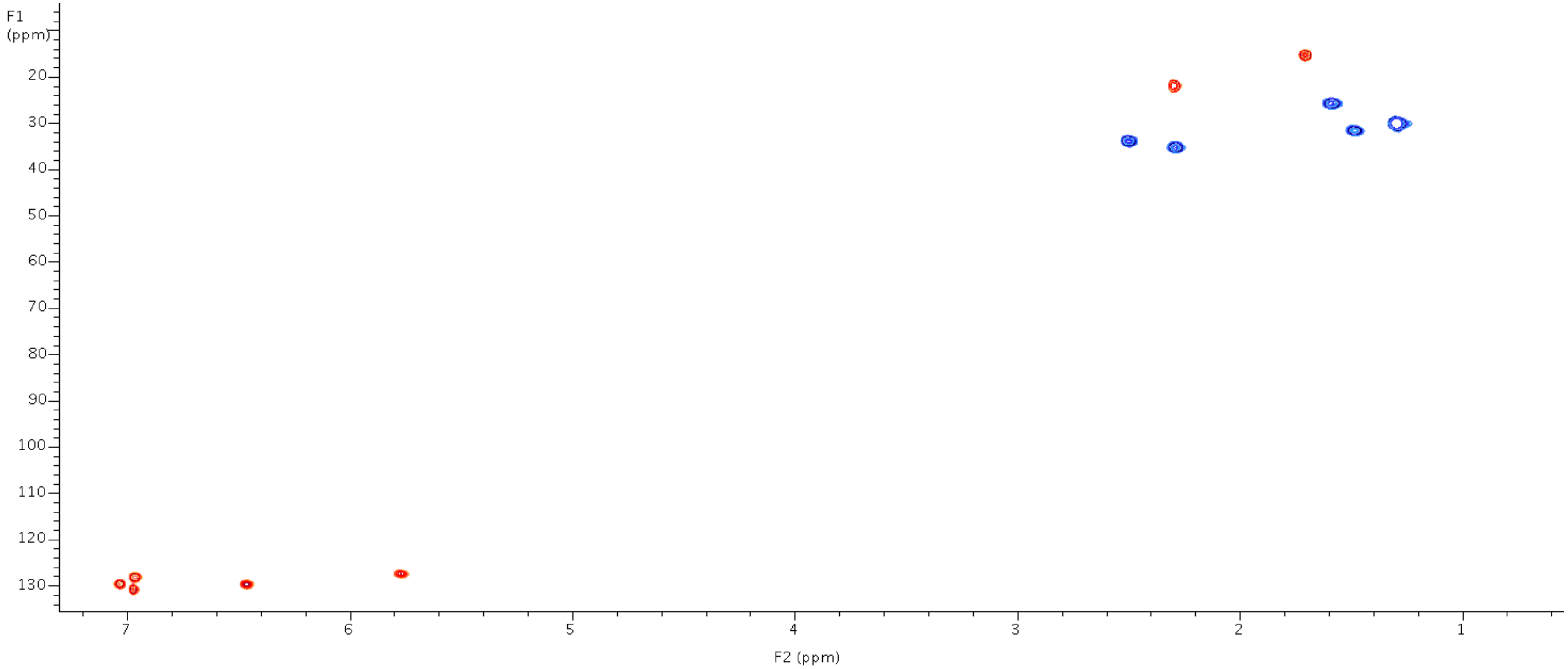

Figure S13. gHMBC (600 MHz, $CDCl_3$) of **2**.

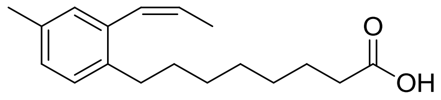

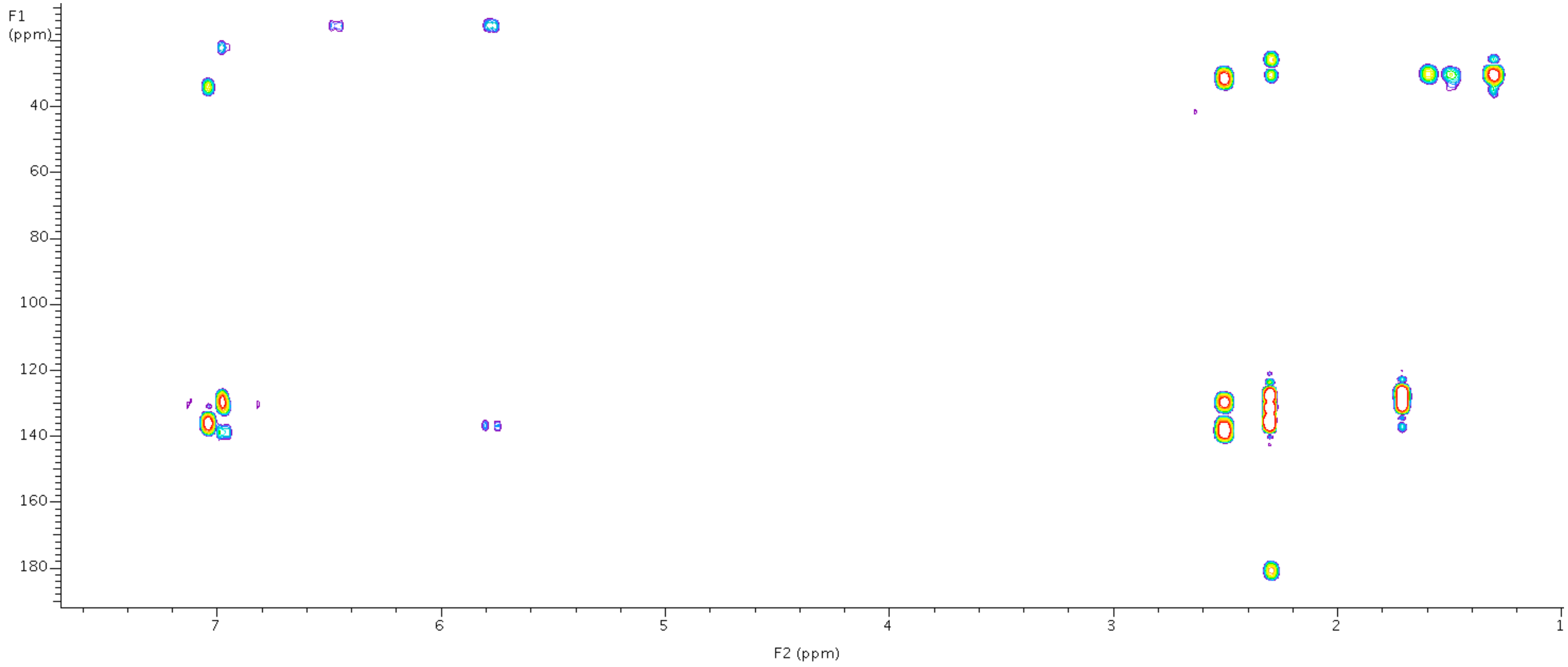

Figure S14. ^{13}C NMR (125 MHz, $CDCl_3$) of ^{13}C-labeled **2**.

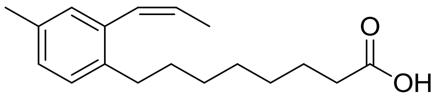

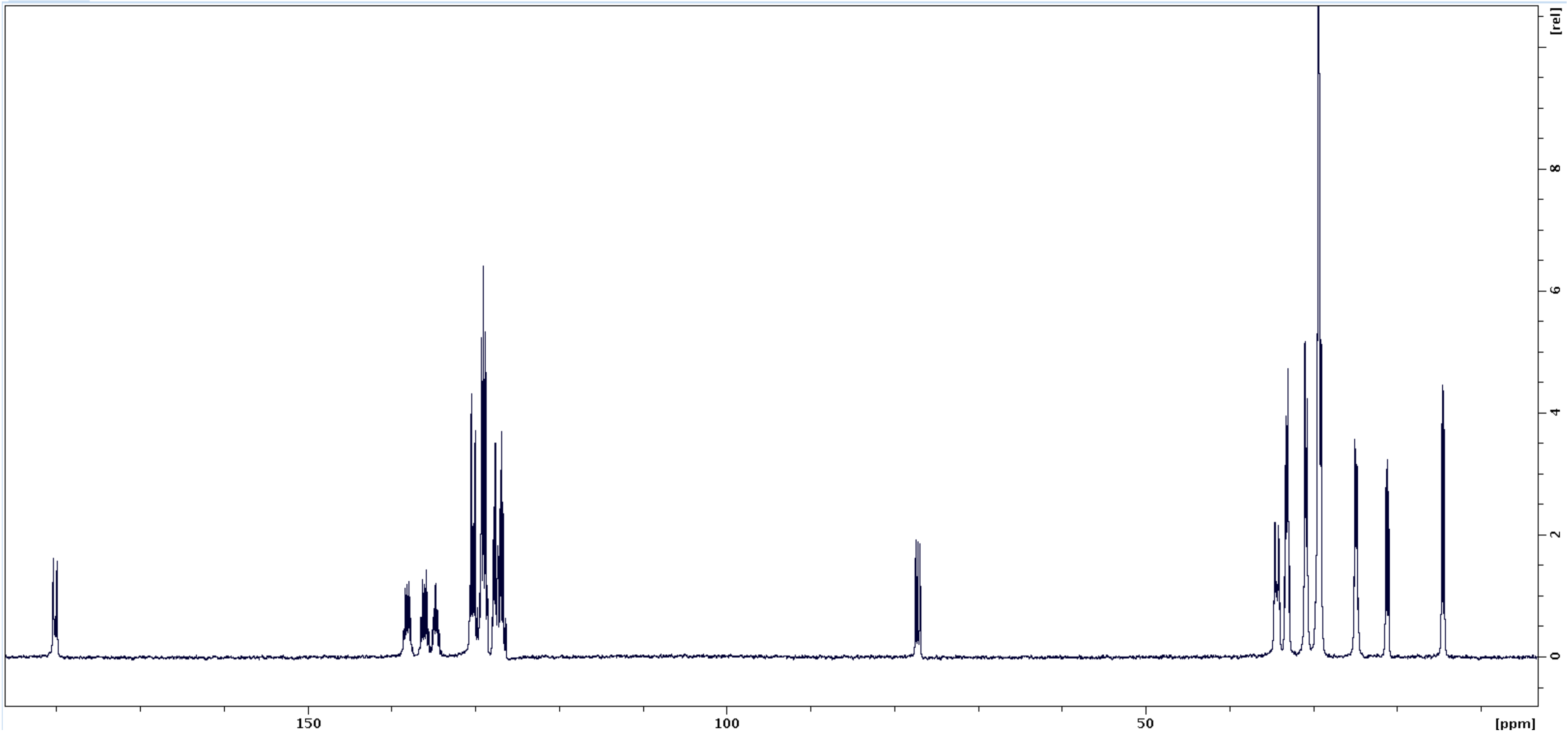

Figure S15. ^{13}C-^{13}C COSY (125 MHz, $CDCl_3$) of ^{13}C-labeled **2**.

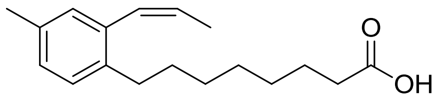

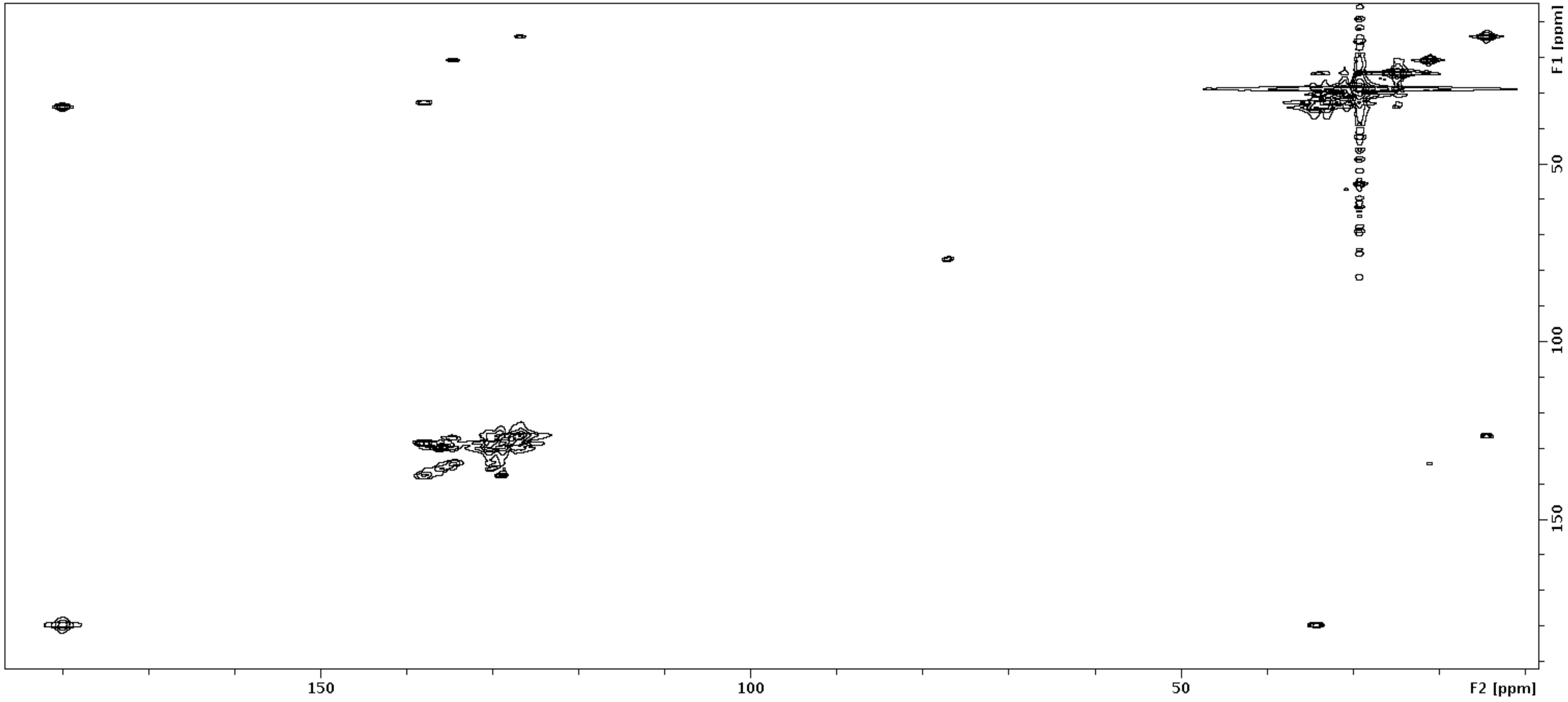

Figure S16. HRMS of **2**.

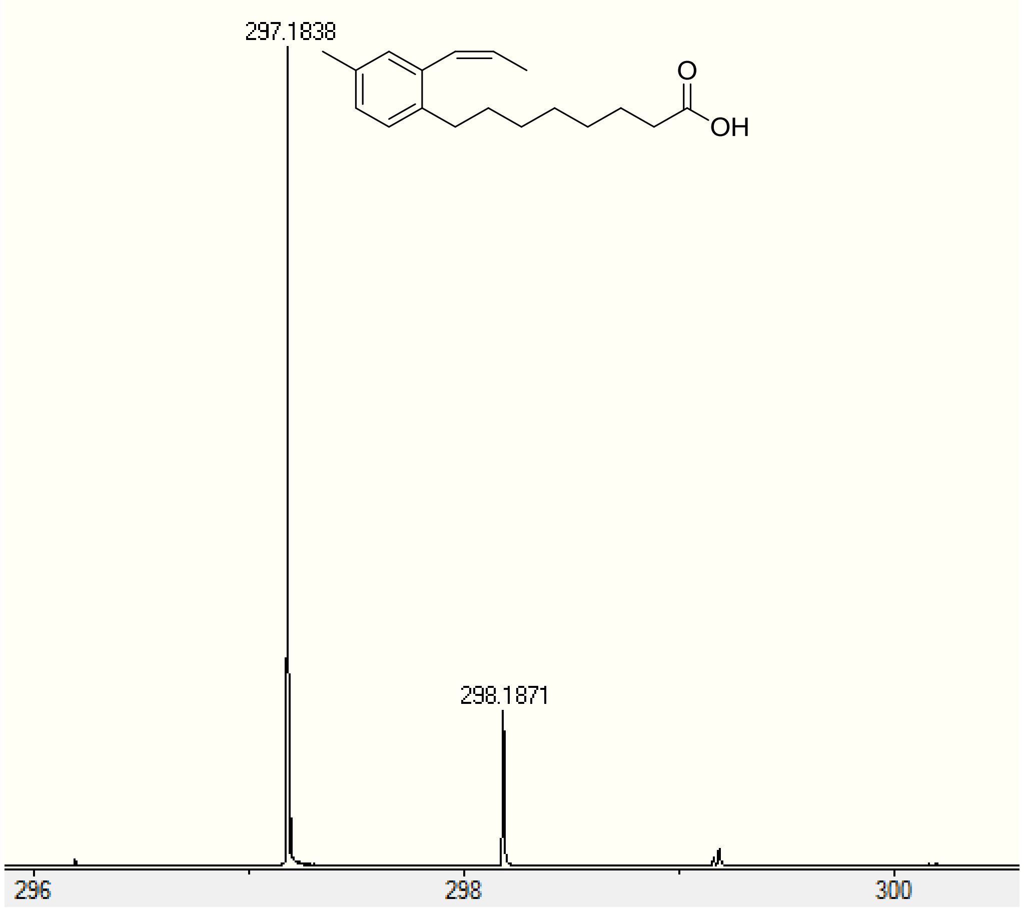

Figure S17. ^{13}C-^{13}C gCOSY calculations.

1. During t_1, each new fid increments t_1 by a dwell time of $dw1 = \frac{1}{sw1}$ (always true for each complex pair of FIDs, independent of type of acquisition, such as States or TPPI, etc.). For a ^{13}C–^{13}C cosy, $sw = sw1 \leq$ 220ppm, which = 125Hz/ppm$\times$220ppm = 27.5kHz. Thus, $dw1$ = 1/27.5kHz = 36.4μs.

2. For J_{CC} ranging 30 to 60 Hz, maximum polarization transfer (largest crosspeak formation) will occur at 1/2J = 33 to 17 ms, respectively.

3. For an absolute-value cosy, where a sinebell or sinebell-squared apodization is applied, maximum crosspeaks (100% of the size of the ^{1}H 1D peak) will occur with a total acquisition time in the indirect dimension, $at1$ = 1/J, because sinebell-type functions are maximum at ½ the FID. So for maximum crosspeaks, the t_1 evolution would be at $at1$ = 66 to 33 ms, for J_{CC} = 30 to 60 Hz.

4. The total acquisition time in the indirect dimension equals the number of increments times the time per increment, or: $at1 = ni \times dw1$ Thus: $ni \equiv \mathrm{TD1} = \frac{at1}{dw1} = \frac{sw1}{J_{CC}}$ = 917 to 458 . Even smaller values (e.g., 256) can be used without much loss in sensitivity, since the crosspeaks intensities will follow something close to sinusoidal behavior close to 90°.

5. Resolution: If ni ≡ TD1 = 400, then sw1/ni = 27500/400 = 69 Hz = 0.55 ppm @ 500 MHz.

Figure S18. ^{13}C-^{13}C COSY pulse program (Bruker).

```
;cosygpqf - modified to have proton decoupling 2June2010
;avance-version (07/04/05)
;2D homonuclear shift correlation
;using gradient pulses for selection
;
;$CLASS=HighRes
;$DIM=2D
;$TYPE=
;$SUBTYPE=
;$COMMENT=

#include <Avance.incl>
#include <Grad.incl>

"d13=4u"

"d11=30m"

"in0=inf1"

"d0=3u"

1 ze
 d11 pl12:f2
2 30m do:f2
 d1 cpd2:f2
3 p1 ph1
 d0
 50u UNBLKGRAD
 p16:gp1
 d16
 p0 ph2
 p16:gp1
 d16
 4u BLKGRAD
 go=2 ph31
 30m do:f2 pl12:f2 mc #0 to 2 F1QF(id0)
exit

ph1=0 2
ph2=0 0 2 2
ph31=0 2

;pl1 : f1 channel - power level for pulse (default)
;p0 : f1 channel - 20 to 90 degree high power pulse
;p1 : f1 channel - 90 degree high power pulse
;p16: homospoil/gradient pulse
;d0 : incremented delay (2D) [3 usec]
;d1 : relaxation delay; 1-5 * T1
;d13: short delay [4 usec]
;d16: delay for homospoil/gradient recovery
;inf1: 1/SW = 2 * DW
;in0: 1/(1 * SW) = 2 * DW
;nd0: 1
;NS: 1 * n
;DS: 16
;td1: number of experiments
;FnMODE: QF

;use gradient ratio: gp 1
; 10
;for z-only gradients:
;gpz1: 10%
;use gradient files:
;gpnam1: SINE.100
;$Id: cosygpqf,v 1.5 2007/04/11 13:34:29 ber Exp $
```

Figure S19. ^{13}C-^{13}C COSY for Varian.

To set up a ^{13}C-^{13}C gCOSY on a Varian spectrometer with VNMR software, complete the following steps:

1. Set up (and acquire) a 1D ^{13}C spectrum with the desired parameters (sweep width, *etc*.) for your particular compound.
2. In the same experiment, set up a gCOSY (type **gCOSY**)
3. Your transmitter nucleus (**tn**) should be **C13**, and decoupler nucleus (**dn**) should be **H1**.
4. Type **dm** = **'nny'**

Set number of transients and increments (**nt**, **ni**) as necessary for your compound

To set up a ^{13}C-^{13}C gCOSY on a Varian spectrometer with VNMRJ software, complete the following steps:

1. Set up (and acquire) a 1D ^{13}C spectrum with the desired parameters (sweep width, *etc*.) for your particular compound.
2. In a different experiment, set up a gCOSY (type **Gcosy**)
3. Change transmitter nucleus (**tn**) to **C13**, and decoupler nucleus (**dn**) should be **H1**.
4. Type **dm** = **'nny'**
5. Change sweep width (**sw**) and **sw1** to sweep width from 1D ^{13}C spectrum.
6. Set number of transients and increments (**nt**, **ni**) as necessary for your compound.

Set remaining parameters as the following:

ACQUISITION		TRANSMITTER		GRADIENTS		PROCESSING	
seqfil	gCOSY	tn	C13	gzlvl1	4237	sb	-0.065
sw	31446.5	sfrq	125.698	gt1	0.001000	sbs	not used
at	0.130	tof	1883.3	gstab	0.000500	fn	8192
np	8192	tpwr	63	hsglvl	4237	2D PROCESSING	
ss	8	pw	11.800	hsgt	0.005000	sb1	-0.003
d1	4.000	DECOUPLER		FLAGS		sbs1	not used
nt	90	dn	H1	hs	nn	proc1	lp
ct	0	dm	nny	sspul	n	fn1	8192
2D ACQUISITION		SPECIAL		SAMPLE			
sw1	31446.5	temp	25.0	date	Oct 29 2012		
ni	140	spin	0	solvent	CD3OD		
PRESATURATION		gain	60	sample			
satmode	n						

Figure S20. (**A**) List of ^{13}C-^{13}C correlations for **1** and **2** and (**B**) ^{13}C-^{13}C COSY (125 MHz, $CDCl_3$) of **2** (aromatic region).

(**A**)

1	2
C1-C2	C1-C2
C2-C3	C2-C3
C3-C4 *	C3-C4
C8-C9	C8-C9
C9-C10	C9-C10 ***
C11-C12	C11-C12
C12-C13	C12-C13
C12-C14	C12-C14
C14-C15	C14-C15
C15-C16 **	C16-C17 ****
C16-C17 **	C17-C18
C17-C18	

* Weak correlation. Confirmed by additional NMR data.
** C15 and C17 overlap. Additional NMR confirmed connectivity.
*** C10 and C16 had similar chemical shifts. Additional NMR confirmed connectivity.
**** Signal partially overlapped. Additional NMR confirmed connectivity.

(**B**)

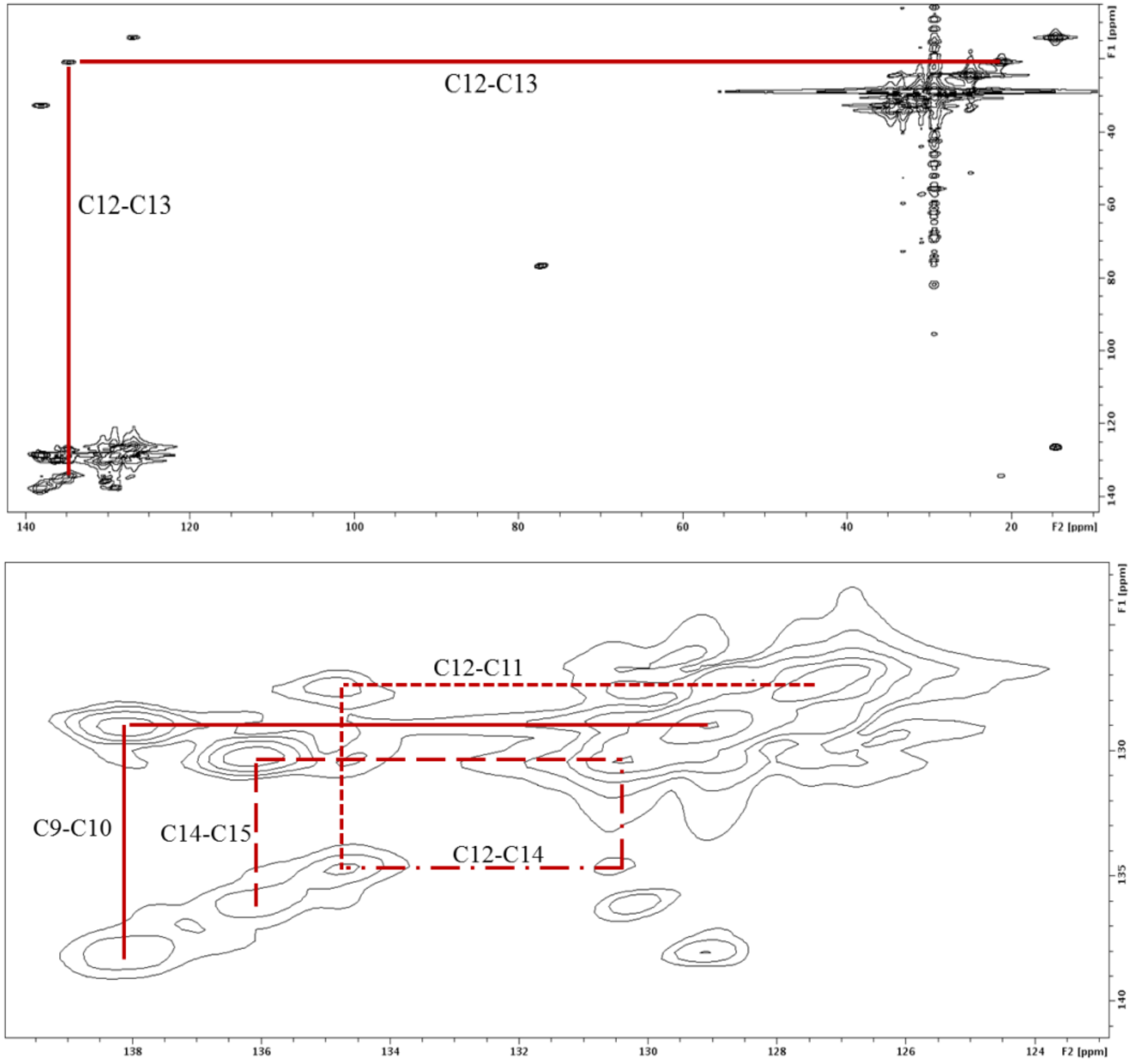

Figure S21. (**A**) ^{13}C-^{13}C COSY (125 MHz, $CDCl_3$) and (**B**) ^{1}H NMR (500 MHz, $CDCl_3$) of ^{13}C-labeled extract from $CHCl_3$ partition.

(**A**)

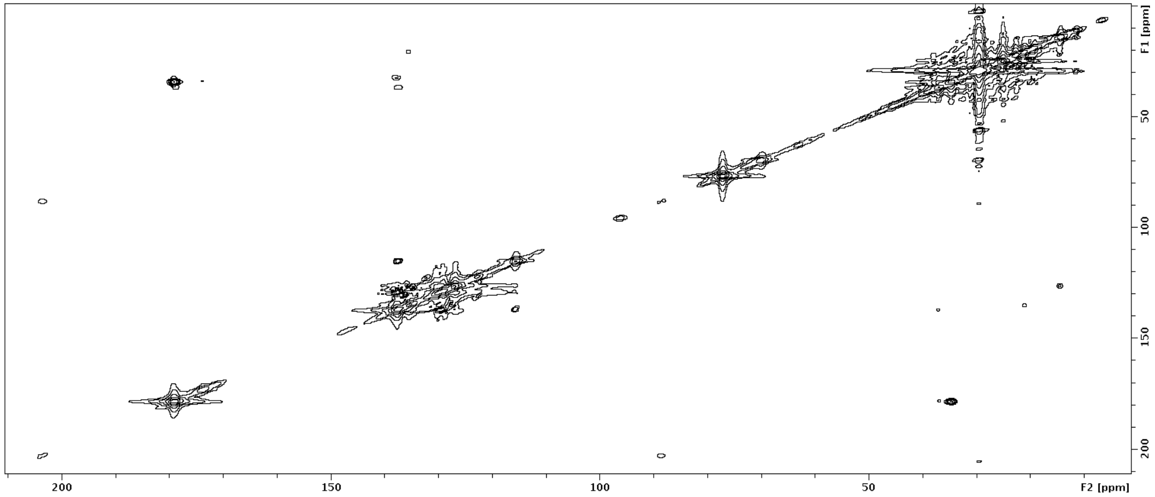

(**B**)

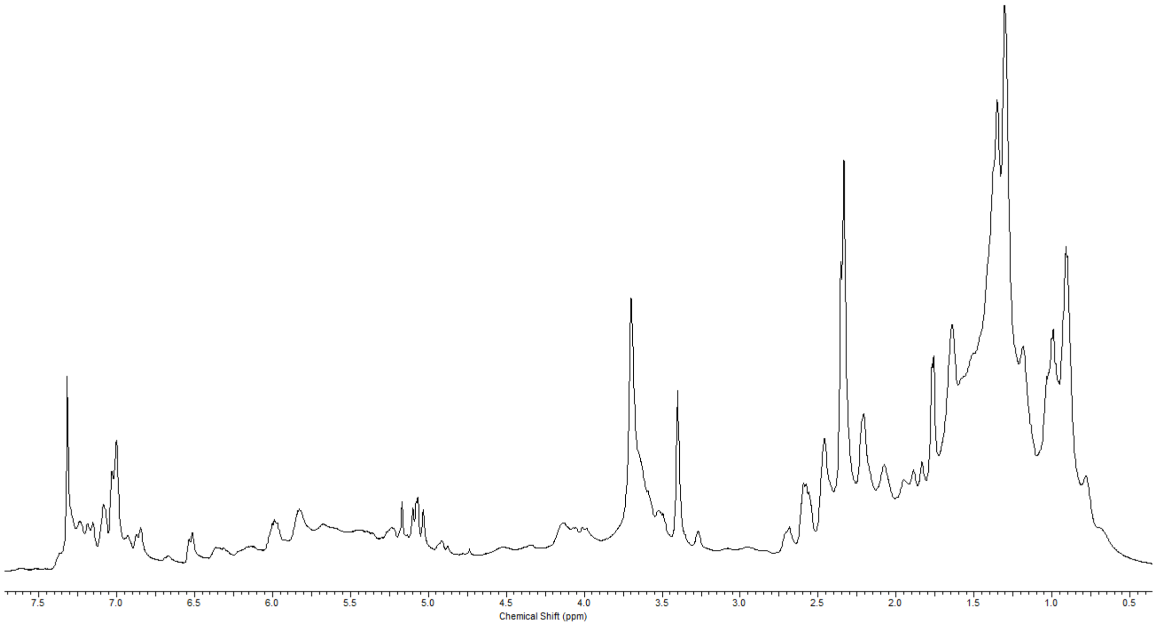

Figure S22. ^{1}H NMR (600 MHz, $CDCl_3$) of **3**.

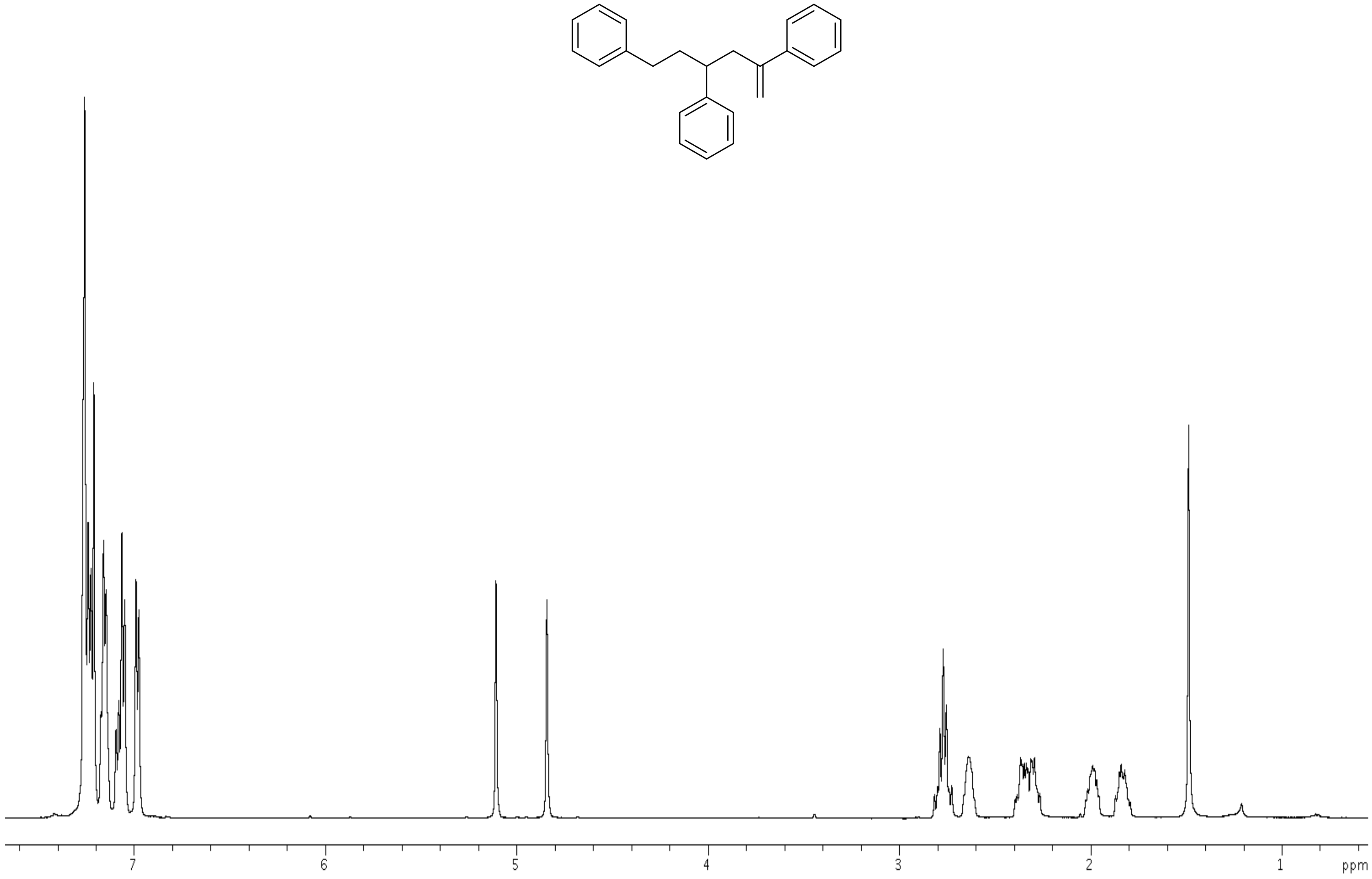

Figure S23. ^{13}C NMR (125 MHz, $CDCl_3$) of **3**.

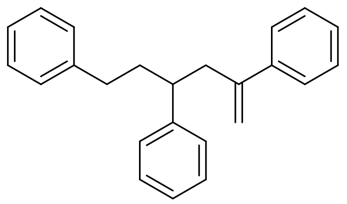

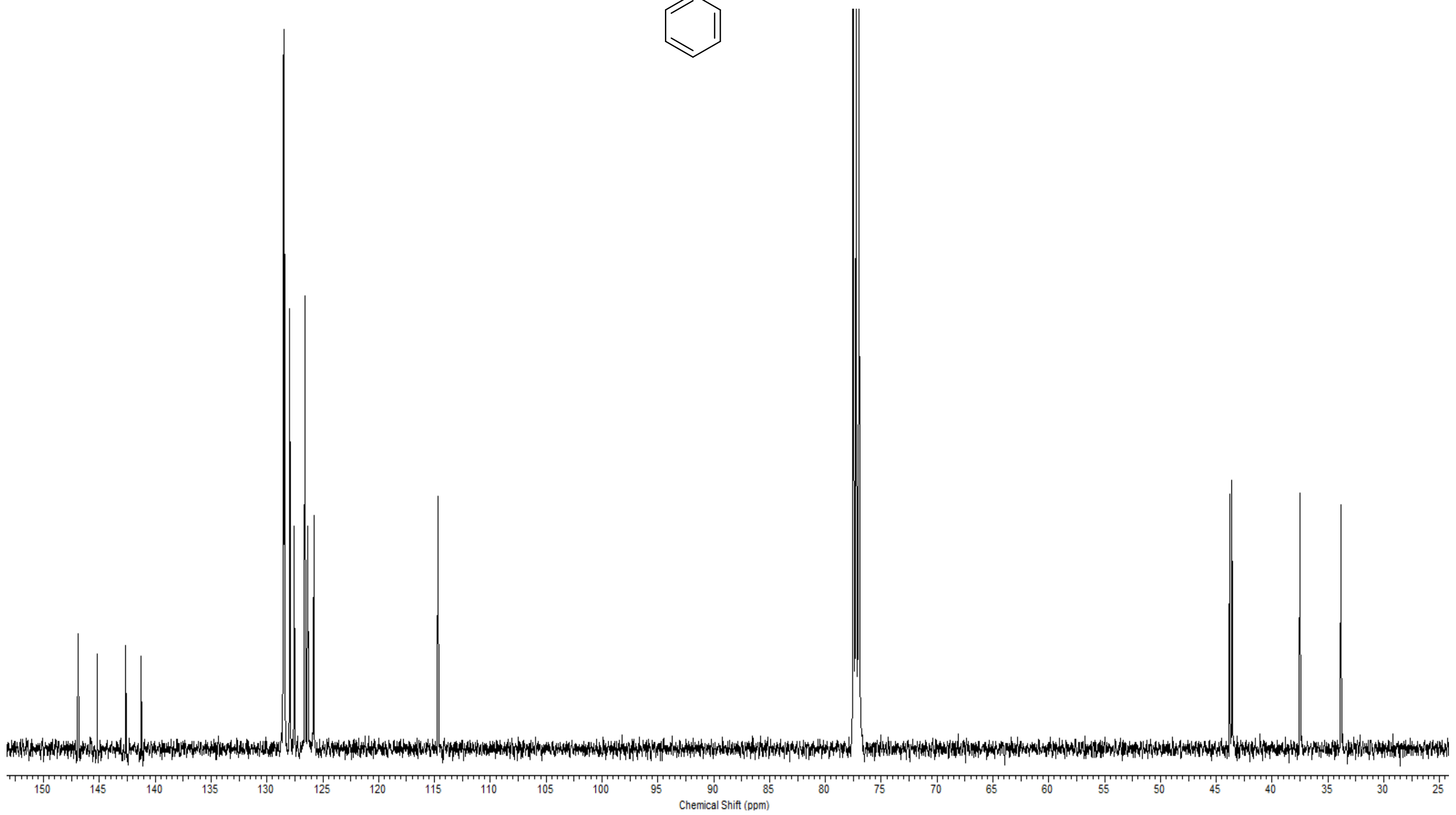

Figure S24. HRMS of **3**.

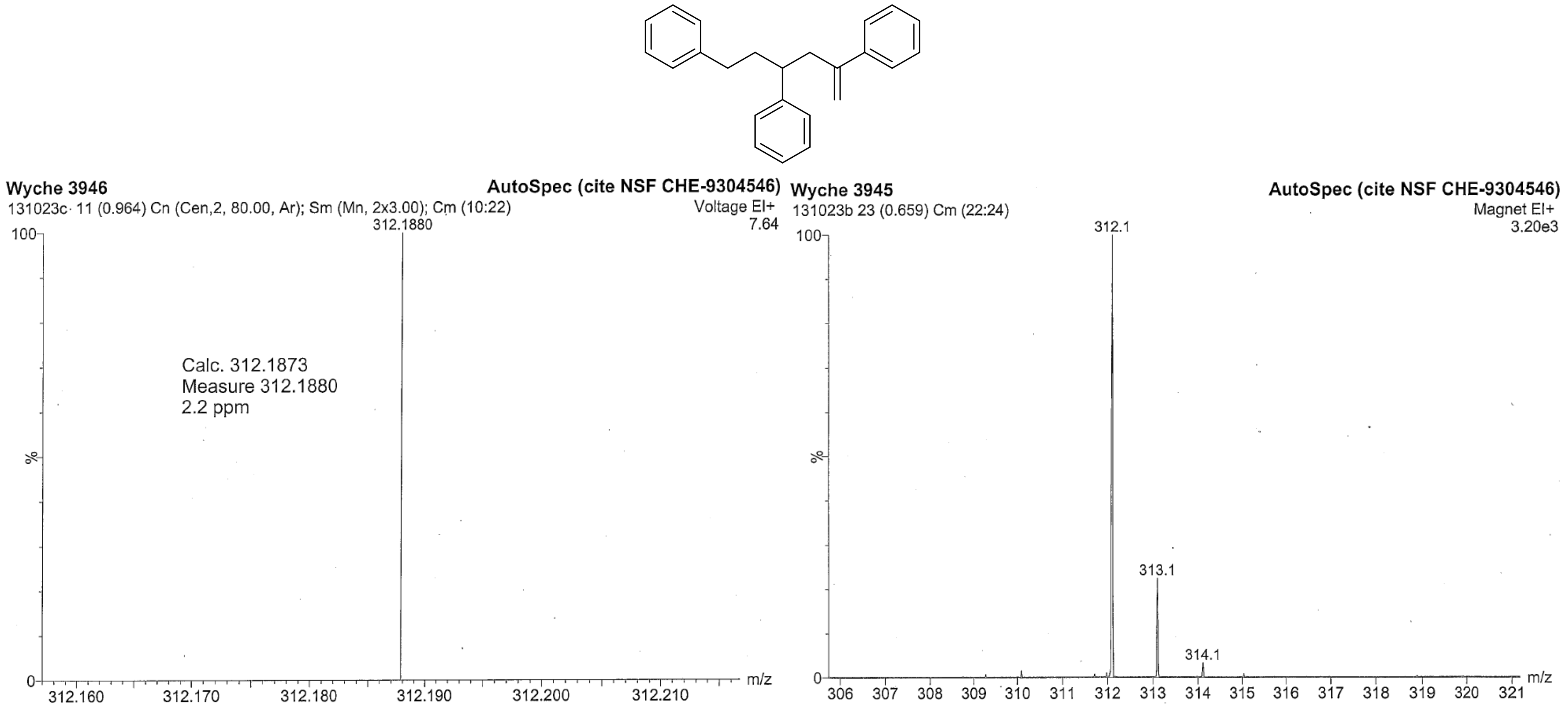

© 2014 by the authors; licensee MDPI, Basel, Switzerland. This article is an open access article distributed under the terms and conditions of the Creative Commons Attribution license (http://creativecommons.org/licenses/by/3.0/).
